# Development and validation of a nomogram for obesity and related factors to detect gastric precancerous lesions in the Chinese population: a retrospective cohort study

**DOI:** 10.3389/fonc.2024.1419845

**Published:** 2024-11-20

**Authors:** Chang’e Shi, Rui Tao, Wensheng Wang, Jinzhi Tang, Zhengli Dou, Xiaoping Yuan, Guodong Xu, Huanzhong Liu, Xi Chen

**Affiliations:** ^1^ Department of Gastroenterology, The First Affiliated Hospital of Anhui Medical University, Hefei, China; ^2^ Department of Gastroenterology, Anhui Public Health Clinical Center, Hefei, China; ^3^ Department of Gastroenterology, The First Affiliated Hospital of Anhui Medical University North District, Hefei, China; ^4^ Department of Psychiatry, Chaohu Hospital of Anhui Medical University, Hefei, China; ^5^ Department of Psychiatry, School of Mental Health and Psychological Sciences, Anhui Medical University, Hefei, China; ^6^ Department of Psychiatry, Anhui Psychiatric Center, Hefei, China; ^7^ Department of Gastroenterology, Chaohu Hospital of Anhui Medical University, Hefei, China; ^8^ Department of Psychiatry, Huizhou NO.2 Hospital, Huizhou, China

**Keywords:** age, body mass index, smoking, triglyceride, associated factors, prediction model, gastric precancerous lesions

## Abstract

**Objectives:**

The purpose of this study was to construct a nomogram to identify patients at high risk of gastric precancerous lesions (GPLs). This identification will facilitate early diagnosis and treatment and ultimately reduce the incidence and mortality of gastric cancer.

**Methods:**

In this single-center retrospective cohort study, 563 participants were divided into a gastric precancerous lesion (GPL) group (n=322) and a non-atrophic gastritis (NAG) group (n=241) based on gastroscopy and pathology results. Laboratory data and demographic data were collected. A derivation cohort (n=395) was used to identify the factors associated with GPLs to develop a predictive model. Then, internal validation was performed (n=168). We used the area under the receiver operating characteristic curve (AUC) to determine the discriminative ability of the predictive model; we constructed a calibration plot to evaluate the accuracy of the predictive model; and we performed decision curve analysis (DCA) to assess the clinical practicability predictive model.

**Results:**

Four –predictors (i.e., age, body mass index, smoking status, and –triglycerides) were included in the predictive model. The AUC values of this predictive model were 0.715 (95% CI: 0.665-0.765) and 0.717 (95% CI: 0.640-0.795) in the derivation and internal validation cohorts, respectively. These values indicated that the predictive model had good discrimination ability. The calibration plots and DCA suggested that the predictive model had good accuracy and clinical net benefit. The Hosmer–Lemeshow test results in the derivation and validation cohorts for this predictive model were 0.774 and 0.468, respectively.

**Conclusion:**

The nomogram constructed herein demonstrated good performance in terms of predicting the risk of GPLs. This nomogram can be beneficial for the early detection of patients at high risk of GPLs, thus facilitating early treatment and ultimately reducing the incidence and mortality of gastric cancer.

## 1 Introduction

Gastric cancer (GC) is a major global health concern because of its poor prognosis and high mortality. It ranks as the fifth most common malignancy in the world and the third leading cause of cancer-related death (1). The age-standardized incidence rates (ASIRs) and age-standardized mortality rates (ASMRs) of GC in the total population, different regions, and countries in Asia showed decreasing trends from 1990 to 2019 (2). In China, there were an estimated 4,824,700 new cancer cases in 2022, 358,700 of which were GC; furthermore, GC remains the fifth most common cancer and the third leading cause of cancer-related deaths in China, despite decreasing trends in the ASIR and ASMR (3). By 2050, it is estimated that there will be 279,707 cases of GC in China, including 122,796 cases of cardiac cancer and 156,911 cases of noncardiac cancer (4). Therefore, it is crucial to develop methods to reduce the global incidence and mortality rates of GC (5, 6).

GC is a multiannual, multistage, and multifactorial disease, and the development of this malignancy has been shown to involve a multistep process from normal gastric mucosa to chronic non-atrophic and atrophic gastritis, intestinal metaplasia, and epithelial dysplasia, and ultimately progressing to GC (7). Various factors can influence the development and progression of GC, including sociodemographic disparities (e.g., age and sex), dietary habits (e.g., high-fat diet and salt intake), bacterial infection (particularly *Helicobacter pylori*), environmental factors (e.g., geographical differences), inherited genes, and gastric precancerous lesions (GPLs) (2, 7–12). Research on anti-gastrointestinal cancer drugs in China is developing rapidly, including studies on PD-1 inhibitors, chimeric antigen receptor (CAR) T-cell therapy, antibody-coupled drugs and bispecific antibodies (13). The most promising approach for preventing GC is to target precancerous lesions (14). Gastric atrophy, intestinal metaplasia, pseudopyloric gland metaplasia, and dysplasia are classified as GPLs during the pathological progression of GC (15–17). Numerous studies have shown that patients with GPLs are at considerable risk of GC (9, 18, 19). A 20-year follow-up study was conducted on patients with GPLs and revealed that incomplete intestinal metaplasia is a significant risk factors for GC. Moreover, anti-H. Pylori therapy was been shown to exert a long-term positive effect on preventing histological progression in a population at high risk of GC (17). Anti-*H. pylori* therapy after endoscopic resection for gastric dysplasia (GPL) has been proven to decrease the risk of developing GC in the long term (20). Wang et al. demonstrated that targeting GPLs (specifically, intestinal metaplasia and epithelial dysplasia) can be an effective intervention for preventing the occurrence of GC (21). The above research clearly revealed that GPLs play a crucial role in the development of GC, and timely identification and early intervention of GPLs are crucial in preventing the development of this malignancy. However, the precise mechanism by which GPLs influence GCs has yet to be fully elucidated, and certain factors associated with GPLs have not yet been identified.

There are numerous predictive models that aim to identify factors associated with GC (22–24). On the basis of the identified associated factors, some studies have developed nomograms with high accuracy for predicting the recurrence of early gastric cancer after ESD (25). These models can include a wide range of factors, such as infection (26–28), metabolism (27, 29), genes and proteins (29–31), and pathological and CT images (28, 32). Gene Ontology (GO) analysis revealed that N1-methyladenosinem1A downstream genes are associated with cell proliferation and clinicopathological parameters in gastrointestinal cancers (33). Previous studies have shown that JMJD3 overexpression is correlated with genetic aberrations and DNA methylation, which are associated with shortened overall survival in patients with GC (34). Despite emerging efforts in the development of anti-gastrointestinal cancer drugs, efforts focused on GC remain insufficient (35). More exploration is needed in the treatment of GC. In recent years, there has been a steady increase in the use of predictive models for GPLs. However, different models emphasize different aspects (36–38). For example, Changzheng Ma et al. developed a machine learning model based on tongue images to screen patients with GPLs, aiming to identify and integrate valuable characteristics of noninvasive medical images related to GPLs (37). Qianyu Zhu et al. conducted a study to identify autoantibodies associated with GPLs via serological proteome analysis, nanoliter−liquid chromatography, and quadrupole time-of-flight tandem mass spectrometry to analyze the potential detection value of GPLs via an enzyme-linked immunosorbent assay (38). A nomogram can assign scores based on predictor values, calculate total scores, and estimate outcome risks in ways that traditional statistical methods cannot. As a common data visualization tool, a prediction model can visually show the relationships among multiple variables and provide us with more comprehensive data analysis results. A prediction model for GPLs could provide valuable guidance to clinicians in identifying high-risk GPL patients and planning more intensive follow-up strategies. Additionally, a prediction model could be used for health guidance, decision-making support, and lifestyle changes for the general population.

In our study, we aimed to construct a predictive model for GPLs on the basis of clinical data to facilitate early interventions for GPLs. The objectives of this study were as follows (1): to construct a nomogram that can accurately predict the probability of GPLs, and (2) to prevent the progression of GPLs to GCs by timely identification and early intervention of individual-associated factors, thereby reducing the overall probability of GC occurrence.

## 2 Methods

### 2.1 Study design and participants

This single-center retrospective cohort study was conducted at the Department of Gastroenterology, The First Affiliated Hospital of Anhui Medical University (North District), from January 2019 to June 2022. To establish a stable and reliable predictive model for GPLs, our study focused on three aspects: (1) identifying the statistically significant risk factors associated with GPLs in the derivation cohort and conducting internal validation in the validation cohort; (2) evaluating the discrimination ability (ROC curve), accuracy (calibration plots), clinical practicability (DCA), and Hosmer–Lemeshow test results in both the derivation and validation cohorts; and (3) conducting a random operation using the original data (numerical variables) and proving that the predictive model also has good consistency among numerical variables. We use the 10 events per variable (EPV) method to obtain a rough estimate of the sample size needed (39). The study was conducted in accordance with the Transparent Reporting of a Multivariable Prediction Model for Individual Prognosis or Diagnosis (TRIPOD) Statement (40).

### 2.2 Data collection

Each enrolled participant underwent gastroscopy and pathology. The clinical data were collected from three different systems: the Hospital Information System (Donghua System), the Image Storage and Communication System (PACS), and the Hospital Electronic Medical Record (HIS). The following data were collected (1). Demographic data included age, sex, body mass index (BMI, kg/m^2^), history of diabetes, history of hypertension, smoking habit, and alcohol use. (2) Fasting biochemical tests and other blood tests were performed to examine C-reactive protein (CRP, mg/l), triglycerides (TG, mmol/l), low-density lipoprotein-cholesterol (LDL-C, mmol/l), uric acid (UA, µmol/l), carcinoembryonic antigen (CEA, ng/ml), and carbohydrate antigen199 (CA199, U/ml). The laboratory tests were conducted within 7 days prior to gastroscopy, and the reference value ranges are shown in **Table 1**. (3) Gastroscopy and pathological examination were performed. Gastroscopy was conducted by a skilled gastroenterologist. The procedure began with an examination of the stomach via white light endoscopy. If any abnormal areas were detected, further investigation and sampling were performed via pigment endoscopy and magnifying endoscopy. Biopsy samples were then sent to the pathology department for standard processing, where a pathologist (junior physician) made a diagnosis. Finally, another pathologist (attending physician or higher) reviewed and aligned the diagnostic opinion to ensure accuracy. (4) Classification data were collected. The pathological findings revealed gastric atrophy, intestinal metaplasia, pseudopyloric metaplasia, and dysplasia, all of which were categorized into the GPL group, and individuals with non-atrophic gastritis were classified into the NAG group. (5) H. pylori was detected via methylene blue staining.

**Table 1:** Clinical baseline characteristics in derivation and validation cohorts.

Variables		Derivation cohort				Validation cohort				p*
		Total	NAG	GPL	p#	Total	NAG	GPL	p#	
		n=395	n=167	n=228		n=168	n=74	n=94		
Sex	Male	228 (57.7)	93 (55.7)	135 (59.2)		107 (63.7)	45 (60.8)	62 (66.0)		
	Female	167 (42.3)	74 (44.3)	93 (40.8)	0.551	61 (36.3)	29 (39.2)	32 (34.0)	0.598	0.220
Age, years old	15-44	44 (11.1)	26 (15.6)	18 (7.9)		26 (15.5)	19 (25.7)	7 (7.45)		
	45-64	226 (57.2)	96 (57.5)	130 (57.0)		89 (53.0)	39 (52.7)	50 (53.2)		
	65+	125 (31.6)	45 (26.9)	80 (35.1)	0.028	53 (31.5)	16 (21.6)	37 (39.4)	0.001	0.339
BMI	Thin/Normal	227 (57.5)	119 (71.3)	108 (47.4)		98 (58.3)	49 (66.2)	49 (52.1)		
	Overweight	136 (34.4)	46 (27.5)	90 (39.5)		61 (36.3)	25 (33.8)	36 (38.3)		
	Obese	32 (8.1)	2 (1.2)	30 (13.2)	<0.001	9 (5.36)	0 (0.0)	9 (9.57)	0.007	0.508
H. pylori	No	293 (74.2)	119 (71.3)	174 (76.3)		121 (72.0)	57 (77.0)	64 (68.1)		
	Yes	102 (25.8)	48 (28.7)	54 (23.7)	0.308	47 (28.0)	17 (23.0)	30 (31.9)	0.268	0.670
Diabetes	No	347 (87.8)	154 (92.2)	193 (84.6)		152 (90.5)	67 (90.5)	85 (90.4)		
	Yes	48 (12.2)	13 (7.8)	35 (15.4)	0.034	16 (9.52)	7 (9.46)	9 (9.6)	1.000	0.451
Hypertension	No	281 (71.1)	122 (73.1)	159 (69.7)		109 (64.9)	51 (68.9)	58 (61.7)		
	Yes	114 (28.9)	45 (26.9)	69 (30.3)	0.544	59 (35.1)	23 (31.1%)	36 (38.3)	0.418	0.170
Smoking	No	280 (70.9)	131 (78.4)	149 (65.4)		112 (66.7)	57 (77.0)	55 (58.5)		
	Yes	115 (29.1)	36 (21.6)	79 (34.6)	0.007	56 (33.3)	17 (23.0)	39 (41.5)	0.018	0.370
Alcohol use	No	303 (76.7)	132 (79.0)	171 (75.0)		129 (76.8)	61 (82.4)	68 (72.3)		
	Yes	92 (23.3)	35 (21.0)	57 (25.0)	0.413	39 (23.2)	13 (17.6)	26 (27.7)	0.176	1.000
CRP	Normal	370 (93.7)	159 (95.2)	211 (92.5)		162 (96.4)	72 (97.3)	90 (95.7)		
	High	25 (6.3)	8 (4.8)	17 (7.46)	0.387	6 (3.6)	2 (2.7)	4 (4.26)	0.695	0.267
TG	Normal	272 (68.9)	133 (79.6)	139 (61.0)		104 (61.9)	51 (68.9)	53 (56.4)		
	High	123 (31.1)	34 (20.4)	89 (39.0)	<0.001	64 (38.1)	23 (31.1)	41 (43.6)	0.133	0.132
LDL-C	Normal	365 (92.4)	158 (94.6)	207 (90.8)		157 (93.5)	71 (95.9)	86 (91.5)		
	High	30 (7.6)	9 (5.4)	21 (9.21)	0.221	11 (6.5)	3 (4.1)	8 (8.51)	0.350	0.795
UA	Normal	274 (69.4)	121 (72.5)	153 (67.1)		110 (65.5)	38 (51.4)	72 (76.6)		
	High	121 (30.6)	46 (27.5)	75 (32.9)	0.303	58 (34.5)	36 (48.6)	22 (23.4)	0.001	0.419
CEA	Normal	376 (95.2)	157 (94.0)	219 (96.0)		160 (95.2)	71 (95.9)	89 (94.7)		
	High	19 (4.8)	10 (6.0)	9 (4.0	0.485	8 (4.8)	3 (4.1)	5 (5.3)	1.000	1.000
CA199	Normal	385 (97.5)	162 (97.0)	223 (97.8)		165 (98.2)	74 (100)	91 (96.8)		
	High	10 (2.5)	5 (3.0)	5 (2.2)	0.749	3 (1.8)	0 (0.0)	3 (3.2)	0.256	0.764

Data was expressed as n (%). NAG, non-atrophic gastritis; GPL, gastric precancerous lesions; p, p-value; BMI, body mass index (Thin/Normal < 24.0 kg/m^2^, Overweight: 24.0-28.0 kg/m^2^, Obese > 28.0 kg/m^2^); H. pylori, helicobacter pylori; CRP, C-reactive protein (Normal: 0-10mg/L, high>10mg/L); TG, triglyceride (Normal: 0-1.7 mmol/L, high>1.7 mmol/L); LDL-C, low-density lipoprotein-cholesterol (Normal: 0-3.62 mmol/L, high>3.62 mmol/L); UA, uric acid (Normal: 0-357umol/l, high>357 umol/l); CEA, carcinoembryonic antigen (Normal: 0-5 ng/ml, high>5 ng/ml); CA199, carbohydrate antigen199 (Normal: 0-37 umol/l, high>37 umol/l).

p# for difference between the NAG and GPL groups in the Derivation and validation cohorts, respectively.

p* for difference between the Derivation cohort and validation cohort.

### 2.3 Statistical analysis

Categorical variables are presented as frequencies and percentages. In the **Supplementary Materials**, numerical variables and measurement data are expressed as the means ± standard deviations (SDs). In the univariate analysis, the chi-square test was used to compare the differences between the GPL group and the NAG group for the categorical variables and counting data. Independent samples t tests or Mann−Whitney U tests were performed to compare quantitative data, as shown in the **Supplementary Materials**. To select predictive factors for the model, we analyzed each variable via univariate analysis and selected variables with p < 0.2 to be entered into the multivariate binary logistic regression model. Age, BMI, smoking status, and hyperlipidemic status were included in the statistical model (p < 0.2), and based on a review of previous literature considering the impact of HP on GPL, HP was also included in the statistical analysis. Finally, statistically significant variables (p < 0.05) were selected as predictive factors for the establishment of the nomogram. The derivation cohort and internal validation cohort were randomly selected at a 7:3 ratio from the total sample. The internal validation of the GPL model was performed via the bootstrap technique (with 1000 resampling bootstraps). To further verify the reliability of the predictive model, we conducted a random operation using the original data (numerical variables) and demonstrated that the model also showed good consistency among the numerical variables. We calculated the AUC to assess the discriminative ability, constructed a calibration plot to evaluate the accuracy of the predictive model, and utilized DCA to assess the clinical practicability. The evaluation of the GPL model’s performance was conducted on both the derivation and validation cohorts. All the statistical analyses were performed via R software version 4.1.2, and p<0.05 (two-tailed) was considered statistically significant.

## 3 Results

### 3.1 Clinical baseline characteristics

A total of 563 participants, comprising 335 males (59.5%) and 228 females (40.5%), were included in this study. A total of 395 participants were included in the derivation cohort, and the validation cohort included 168 participants recruited from January 2019 to June 2022. Both the derivation cohort and the validation cohort consisted of patients with NAGs (167 and 74, respectively) and GPLs (228 and 94, respectively). No significant differences in clinical baseline demographic characteristics were observed between the derivation cohort and the validation cohort, as shown in **Table 1**.

### 3.2 Univariate logistic regression analysis for identifying the risk factors for with GPL

In the derivation cohort, 6 clinical indicators were statistically significant in the univariate analysis: age (45-64 years: p=0.045; 65+ years: p=0.009), BMI (overweight and obese: all, p<0.001), history of diabetes (p=0.026), smoking (p=0.005), and TG (p<0.001) (**Table 2**). These indicators were then entered into a multivariate logistic regression model. Additionally, the p value for the HP comparison between the GPL group and the NAG group in the derivation cohort was 0.257, which was close to 0.20. Because HP is a crucial factor in clinical disease, it was also included in the multivariate logistic regression model.

**Table 2:** Univariate and multivariate logistic regression analysis of risk factors for GPL based on the derivation cohort.

Variables	Univariate analysis				Multivariate analysis			
	Coef.	OR	95%CI	p	Coef.	OR	95%CI	p
Female (ref. Male)	-0.144	0.866	0.578-1.297	0.484				
Age (ref. 15-44 years old)								
45-64	0.671	1.956	1.021-3.821	0.045	0.749	2.114	1.039-4.415	0.042
65+	0.943	2.568	1.280- 5.255	0.009	1.063	2.894	1.351-6.372	0.007
BMI (ref. Thin/Normal)								
Overweight	0.768	2.156	1.393-3.367	<0.001	0.718	2.020	1.299-3.265	0.002
Obese	2.805	16.528	4.835-103.685	<0.001	2.865	17.551	5.030-111.242	<0.001
H. pylori (ref. No)	-0.262	0.769	0.489-1.212	0.257				
Diabetes (ref. No)	0.765	2.148	1.124-4.345	0.026				
Hypertension (ref. No)	0.163	1.177	0.757-1.841	0.473				
Smoking (ref. No)	0.657	1.929	1.227-3.077	0.005	0.495	1.640	1.011-2.688	0.047
Alcohol use (ref. No)	0.229	1.257	0.782-2.041	0.348				
CRP (ref. Normal)	0.471	1.601	0.694-4.011	0.286				
TG (ref. Normal)	0.918	2.505	1.591-4.010	<0.001	0.923	2.516	1.555-4.136	<0.001
LDL-C (ref. Normal)	0.577	1.781	0.817-4.193	0.161				
UA (ref. Normal)	0.254	1.289	0.835-2.006	0.255				
CEA (ref. Normal)	-0.438	0.645	0.251-1.637	0.352				
CA199 (ref. Normal)	-0.320	0.726	0.199-2.651	0.618				

Coef., coefficient; OR, Odds Ratio; 95%CI, 95% Confidence Interval; ref., reference.

### 3.3 Multivariate logistic regression analysis and construction of a nomogram

Multivariate logistic regression analysis revealed that age, BMI, and TG were independent predictors of GPL risk. Adding the smoking variable to the regression model improved the overall fit of the model, resulting in an increased NRI value of 0.130. Finally, four valuable risk factors (age, BMI, smoking, and TG) were selected as predictors to establish the predictive model (**Table 2**), and the Hosmer–Lemeshow test result in the derivation cohort for the predictive model was favorable (p=0.774).

**Figure 1:**
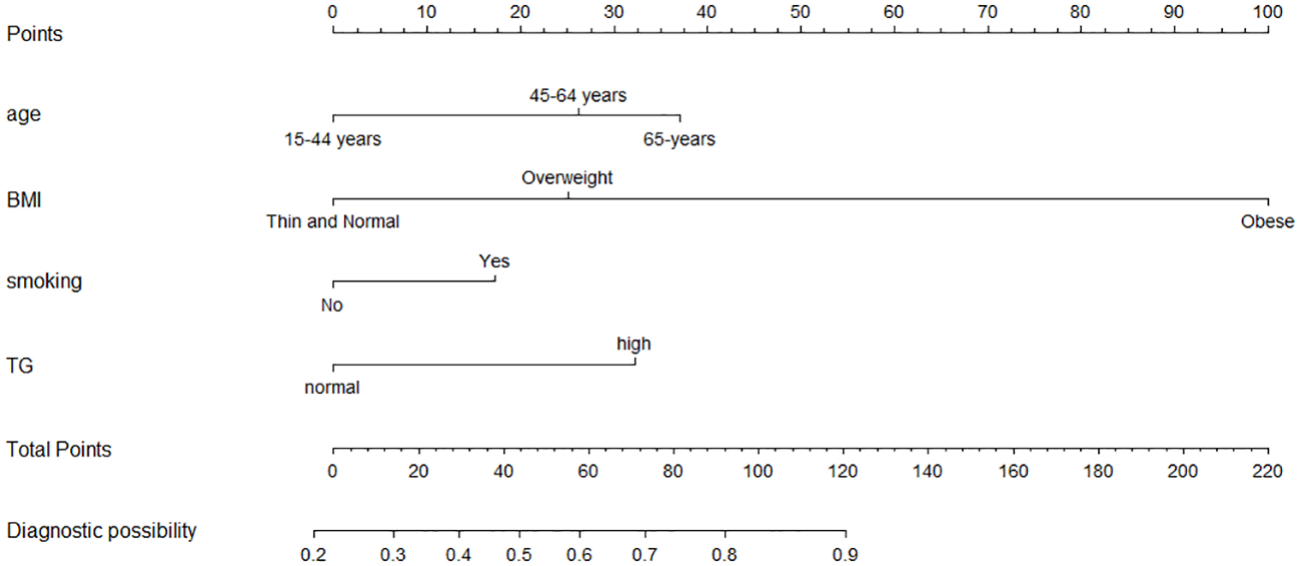
Nomogram model for the risk prediction of GPL. Age, BMI, smoking and TG. Each categorical variable was scored on a scale of 0–100, and the total score for predicting GPL was obtained by summing up the scores corresponding to each categorical variable. Different total score corresponded to different probability of categorical.

A nomogram was constructed based on the multivariate logistic regression model (**Figure 1**). To provide more options for clinical use, we listed the formula for the predictive model here: logit(P) =0.749*age(45-64 years old)(or 1.063*age (65 + years old)) + 0.718*BMI(overweight)(or 2.865*BMI (obese)) + 0.495*smoking + 0.923*TC - 1.253; P = 1/[1+e^−(0.749*age(45-64 years old)(or 1.063*age(65+ years old)) + 0.718*BMI (overweight)(or 2.865*BMI (obese)) + 0.495*smoking + 0.923*TC - 1.253)]^.

### 3.4 Evaluating the performance of the predictive model built based on the derivation cohort

**Figure 2:**
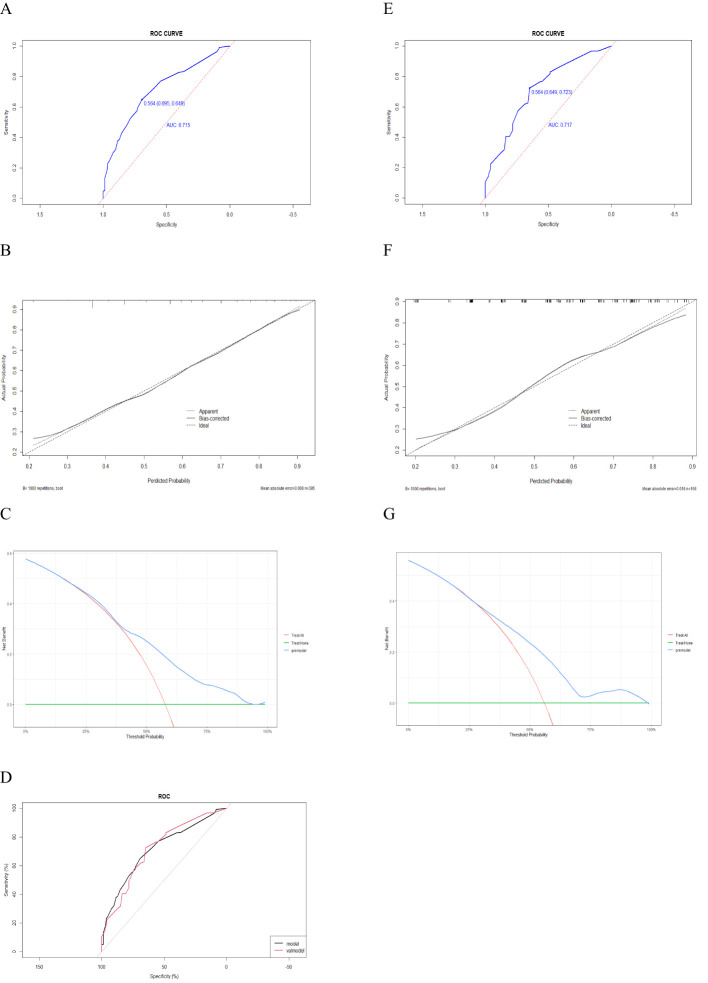
ROC curves, calibration plots, DCA, and DeLong's test of the prediction model in the derivation cohort and validation cohort. **(A, E)**, **(B, F)**, **(C, G)** represent the ROC curve, calibration curve, and DCA in the derivation and validation cohort, respectively. **(D)** represent DeLong's test for ROC curves between the derivation and validation cohorts; Calibration plots showed ideal (100% agreement) curves, the apparent (actual) and bias-corrected (adjusted) (with 1000 resampling bootstrap). The y-axis represents the actual probability of the GPL model, and the x-axis represents the predicted probability of the GPL model. Clinical decision curve: the y-axis represents the net benefit of GPL, while the x-axis represents threshold probability of GPL. The green line stands for the net benefit for the predict-none-patients as GPL, the red line stands for the net benefit for the predict-all-patients as GPL, and the blue line represents net benefit for the GPL model.

ROC curve analysis revealed that the AUC of this predictive model in the derivation cohort was 0.715 (95% CI: 0.665-0.765). The maximum YDI was 0.649, and the corresponding cutoff value was 0.564, demonstrating good discrimination of the predictive model (**Figure 2A**).

The calibration curve showed good concordance between the actual observations and the nomogram-predicted probabilities in the derivation cohort (**Figure 2B**). The decision curve of this predictive model revealed that this nomogram was effective in clinical practice (**Figure 2C**).

### 3.5 Internal validation and other validation

The bias-corrected AUC derived from internal validation via the bootstrap method was 0.717 (95% CI: 0.640–0.795). The maximum YDI was 0.723, and the corresponding cutoff value was 0.564, similar to the AUC (0.715, 95% CI: 0.665-0.765) calculated in the derivation cohort (**Figures 2A, E**). The Hosmer–Lemeshow test results in the validation cohort for the predictive model were good (p=0.468). The calibration plot demonstrated good consistency between the actual and nomogram predictions in the internal validation cohort (**Figure 2F**). The DCA outperformed both extreme lines across a wide range of threshold probabilities, which also demonstrated the good performance of the nomogram in clinical practice in internal validation (**Figure 2G**). DeLong’s test for ROC curves between the derivation and validation cohorts revealed no significant difference (p = 0.960) (**Figure 2D**).

To further verify the reliability of the predictive model, we conducted a random operation using the original data (numerical variables) and proved that the model also has good consistency among the numerical variables. In the new derivation and validation, ROC curve analysis revealed that the AUCs of this predictive model were 0.711 (95% CI: 0.661-0.762) and 0.701 (95% CI: 0.622-0.780), respectively. The Hosmer–Lemeshow test results for the new derivation and validation were good (p=0.111, p=0.220). For more details, please refer to the **Supplementary Materials**.

The calibration plots revealed ideal (100% agreement) curves and apparent (actual) and bias-corrected (adjusted) (1000 resamples) curves. The y-axis represents the actual probability of the GPL model, and the x-axis represents the predicted probability of the GPL model. Clinical decision curve: the y-axis represents the net benefit of the GPL, whereas the x-axis represents the threshold probability of the GPL. The green line represents the net benefit for the predict-no-patients as GPL, the red line represents the net benefit for the predict-all-patients as GPL, and the blue line represents the net benefit for the GPL model.

## 4 Discussion

Numerous studies have confirmed the close relationship between GPLs and GC from various perspectives (41, 42), and patients with GPLs are at considerable risk of GC (9, 18, 19). Gastric atrophy, intestinal metaplasia, pseudopyloric gland metaplasia, and dysplasia are generally recognized as GPLs, and targeting GPLs can be an effective intervention to prevent the occurrence of GC (20, 21). Therefore, developing a simple and convenient predictive model for GPLs, rather than relying on invasive gastroscopy procedures, can aid in early intervention for managing GPLs and effectively reduce the incidence of GC. In this study, we developed and validated a nomogram to preliminarily predict the risk of GPLs. The predictive model includes four predictors: age, BMI, smoking status, and TG.

### 4.1 Age

Age is a significant risk factor for the development of both cancerous and precancerous conditions (14, 37, 43–46). Hai-Fan Xiao et al. conducted a study on risk factors for upper GPLs in non-high-incidence areas and reported that age is the most significant contributor to the risk of developing GPLs (47). A follow-up study of 16,764 French patients who underwent upper endoscopy with gastric biopsies revealed that the severity of precancerous lesions tends to increase with age (48). According to a study conducted by Romańczyk M et al., individuals who are 40 years or older are more likely to be diagnosed with precancerous conditions than patients younger than 40. Furthermore, the risk of dysplasia begins to increase at age 55, whereas the risk of cancer increases at age 60 (43). The prevalence rate of chronic atrophic gastritis is relatively high in China and tends to increase with age (49). A large population-based study in China (27,094 participants) revealed that age was an essential sociodemographic risk factor for GC and GPL and that age was associated with all GPLs, with relative risks ranging from 1.01 to 1.13 (12). In the present study, the age of the GPL group was greater than that of the NAG group in both the derivation and validation cohorts. Among the 322 GPL patients in our study, 180 (55.9%) were between 45 and 64 years old, and 117 (36.3%) were over 65 years old. These proportions align with the findings of the previous study, indicating the need for increased focus on GPL screening for individuals over 45 years old.

### 4.2 Smoking

Among the various habits that play a role in GPL development, the impact of smoking has been considered (50). Smoking is known to be associated with a greater risk of recurrence and death from GC (51, 52), and this association also seems to be evident in GPLs (16, 45). Smoking can increase gastric secretion and has been linked to increased levels of plasma pepsinogen I, which is a marker for GPLs (53). Our findings revealed that smokers are more likely to have GPLs (OR=1.640), which is consistent with the findings of previous studies (16, 53). Studies have shown that smoking is strongly linked to a greater risk of developing GPLs in China, particularly among Han Chinese individuals. The risk was significantly greater for individuals who smoked fewer than 10 cigarettes per day (OR = 5.24), between 11 and 20 cigarettes per day (OR = 8.19), and those who smoked 21 or more cigarettes per day (OR = 7.07) (16). Early research conducted in rural China has also demonstrated a clear link between smoking and the development of GPLs. Moreover, this association becomes more pronounced with greater daily consumption and a longer duration of smoking (14). A microsimulation model suggested that the relative decline in cancer incidence was accelerated by 7% due to lower rates of smoking initiation and higher rates of smoking cessation observed after the 1960s (54). Currently, implementing tobacco control measures among individuals with GPLs is widely recognized as crucial (16), and these measures play a significant role in preventing the onset and progression of GPLs.

### 4.3 MBI and TG

Many studies have confirmed that TG is a risk factor for GPLs, and this predictor is significantly more common in overweight and obese people (55). Overweight and obese individuals often have higher levels of TG (56, 57), which is also known to be a risk factor for GPL and GC and is associated with alterations in the gut microbiota (55). Li Tian et al. demonstrated that obesity promotes diethylnitrosamine-induced precancerous lesions by inducing M1 macrophage polarization and angiogenesis in mice, which may involve obesity and inflammation (58). Our study revealed that the risk of GPLs was 2.020 times greater in overweight individuals and 17.551 times greater in obese individuals than in thin or normal individuals. Additionally, individuals with high TG levels have a 2.516-fold greater risk of GPLs than do individuals with normal TG levels. Combined with our previous findings, we speculate that overweight and obese people have higher TG levels, which may be closely related to their diet.

Many studies have reported strong correlations between TG, BMI, and dietary factors (59), and these findings are also supported by studies on GC, GPL, and dietary factors (14, 44, 52, 59). Seiya Arita M.S. et al. reported that a high-fat diet could cause severe microbiota disorders in the stomachs of mice. Changes in the microbiome are accompanied by increased gastric leptin, leading to intestinal metaplasia. This process involves the gastric leptin signaling pathway regulating the microflora of the gastric mucosa (55), and some domestic studies also support the above results (14, 47). Other dietary studies have suggested that consuming fruits and vegetables may effectively reduce the risk of GC, and adhering to a healthy plant-based diet was also found to be negatively correlated with the occurrence of GPLs. Higher consumption of healthy plant-based foods, such as vegetables, fruits, whole grains, and algae, is associated with increased intake of vitamins, dietary fiber, and phytochemicals. These nutrients contribute to the prevention of gastric mucosal lesions by providing anti-inflammatory and antioxidant benefits (59). Therefore, prevention of GPLs can potentially be achieved through dietary intervention. This involves increasing the consumption of fresh fruits and vegetables while limiting an unhealthy plant-based diet. Additionally, making lifestyle changes such as engaging in more regular exercise can also help reduce the risk of developing GPLs (16, 47, 52). In future screening strategies, populations that are overweight, obese, or on a high-fat diet should be identified as important groups for screening GPLs. Health guidance should be provided to individuals who have a higher recommended frequency of gastrointestinal examinations than that of the general population.

### 4.4 Other associated factors

Studies have shown that *H. pylori* infection causes chronic active gastritis, which can progress to atrophic gastritis, intestinal metaplasia, and GPL (low-grade and high-grade dysplasia), ultimately resulting in the development of GC (60–62). *H. pylori* infection is prevalent and is a significant contributor to the occurrence of GC (63). A population-based cohort study involving 69,722 patients with gastric dysplasia revealed that *H. pylori* treatment was associated with a reduced risk of developing GC. In particular, this treatment has a significant protective effect against the development of late-onset GC (20). Many studies have also confirmed the relationship between *H. pylori* and GPLs (64–66). *H. pylori* cytotoxin-associated gene A status is crucial for detecting GPLs (65). However, the predictive model of this study did not reveal any relationship between *H. pylori* and GPLs, which may be attributed to the following. 1) In our study, the subjects were divided into two groups on the basis of pathological findings: the NAG group and the GPL group. During clinical examinations, many individuals do not undergo pathological examinations due to normal findings under gastroscopy. This resulted in a greater number of individuals in the GPL group than in the NAG group, surpassing the figures reported in other studies (48). 2) Smoking has been suggested to possibly decrease the risk of *H. pylori* infection. This theory is based on the assumption that smoking could increase acid production and pepsin secretion, thereby providing some level of protection for the gastric mucosa against *H. pylori* infection (16). Notably, this study did not find any evidence of *H. pylori* infection, which could be explained by this assumption.

In our study, we performed internal validation to assess the performance of the prediction model in terms of discrimination, calibration, and clinical applicability. The results showed that the model performed effectively. Moreover, this prediction model only includes four easily obtainable predictors in clinical practice, making it highly manageable. The aim of constructing this prediction model is to identify individuals who are at risk of developing GPLs and encourage them to undergo early gastroscopy for timely detection and intervention.

### 4.5 Limitations

The limitations in this study should be considered when interpreting the results. First, this was a single-center retrospective study, and the data collected may have potential bias related to changes in sample size and structure, thus explaining the lack of positive findings for certain important indicators, such as *H. pylori*. Second, because this study was retrospective, it was not possible to include all relevant measures mentioned in the references. As a result, some clinically significant measures may have been overlooked. Therefore, future research should focus on revising the prediction model. Third, owing to sample size limitations, we were unable to classify patients with GPLs in greater detail, which hindered our ability to identify high-risk patients more accurately. Finally, conducting a multicenter, randomized controlled and large-sample validation study based on the model is crucial and may contribute to uncovering new findings. This helps improve the accuracy of the model and its applicability.

## 5 Conclusions

This study developed a preliminary nomogram that is reliable and accurate with respect to predicting the risk of GPLs. The model can help with the early detection of patients at high risk for GPLs and reduce the incidence and mortality of gastric cancer by actively intervening in patients with GPLs. In the future, it will be crucial to include more GPL-related factors in the statistical analysis to improve the model. Furthermore, the model should be externally validated in larger sample sizes from multiple centers to further enhance its effectiveness.

## Data Availability

The original contributions presented in the study are included in the article/**Supplementary Material**. Further inquiries can be directed to the corresponding authors.
