# Supplementary material for: Development and validation of a nomogram for obesity and related factors to detect gastric precancerous lesions in the Chinese population: a retrospective cohort study
Source: Front Oncol. 2024 Nov 20;14:1419845. doi: 10.3389/fonc.2024.1419845 (PMC11614725; doi:10.3389/fonc.2024.1419845)
Supplement: Supplementary file 1 [file Table1.docx]

Table 1 Clinical baseline characteristics in development and validation cohorts.

| Variables | Development cohort | | | | Validation cohort | | | |  |
| --- | --- | --- | --- | --- | --- | --- | --- | --- | --- |
|  | Total | NAG | GPL | p# | Total | NAG | GPL | p# | p* |
|  | n=395 | n=166 | n=229 |  | n=168 | n=75 | n=93 |  |  |
| Sex, n (%) |  |  |  | 0.523 |  |  |  | 0.726 | 0.112 |
| Male | 244 (61.8) | 99 (59.6) | 145 (63.3) |  | 91 (54.2) | 39 (52.0) | 52 (55.9) |  |  |
| Female | 151 (38.2) | 67 (40.4) | 84 (36.7) |  | 77 (45.8) | 36 (48.0) | 41 (44.1) |  |  |
| Age, years | 57.8 (12.0) | 54.8 (12.8) | 60.0 (11.0) | <0.001 | 57.8 (12.1) | 56.2 (12.6) | 59.2 (11.5) | 0.112 | 0.997 |
| BMI (kg/m^2^) | 23.6 (3.4) | 22.7 (2.6) | 24.2 (3.9) | <0.001 | 23.1 (3.3) | 22.4 (2.6) | 23.7 (3.7) | 0.005 | 0.147 |
| H. pylori, n (%) |  |  |  | 1.000 |  |  |  | 0.815 | 0.408 |
| No | 286 (72.4) | 120 (72.3) | 166 (72.5) |  | 128 (76.2) | 56 (74.7) | 72 (77.4) |  |  |
| Yes | 109 (27.6) | 46 (27.7) | 63 (27.5) |  | 40 (23.8) | 19 (25.3) | 21 (22.6) |  |  |
| Diabetes, n (%) |  |  |  | 0.252 |  |  |  | 0.162 | 0.451 |
| No | 347 (87.8) | 150 (90.4) | 197 (86.0) |  | 152 (90.5) | 71 (94.7) | 81 (87.1) |  |  |
| Yes | 48 (12.2) | 16 (9.6) | 32 (14.0) |  | 16 (9.5) | 4 (5.3) | 12 (12.9) |  |  |
| Hypertension, n (%) |  |  |  | 0.603 |  |  |  | 0.362 | 1.000 |
| No | 274 (69.4) | 118 (71.1) | 156 (68.1) |  | 116 (69.0) | 55 (73.3) | 61 (65.6) |  |  |
| Yes | 121 (30.6) | 48 (28.9) | 73 (31.9) |  | 52 (31.0) | 20 (26.7) | 32 (34.4) |  |  |
| Tobacco use, n (%) |  |  |  | 0.006 |  |  |  | 0.021 | 0.268 |
| No | 269 (68.1) | 126 (75.9) | 143 (62.4) |  | 123 (73.2) | 62 (82.7) | 61 (65.6) |  |  |
| Yes | 126 (31.9) | 40 (24.1) | 86 (37.6) |  | 45 (26.8) | 13 (17.3) | 32 (34.4) |  |  |
| Alcohol use, n (%) |  |  |  | 0.212 |  |  |  | 0.517 | 0.317 |
| No | 298 (75.4) | 131 (78.9) | 167 (72.9) |  | 134 (79.8) | 62 (82.7) | 72 (77.4) |  |  |
| Yes | 97 (24.6) | 35 (21.1) | 62 (27.1) |  | 34 (20.2) | 13 (17.3) | 21 (22.6) |  |  |
| CRP (mg/l) | 3.6 (9.2) | 2.5 (6.9) | 4.3 (10.5) | 0.040 | 2.3 (4.8) | 2.0 (2.6) | 2.5 (6.1) | 0.468 | 0.028 |
| TG (mmol/l) | 1.6 (1.0) | 1.4 (0.7) | 1.8 (1.1) | <0.001 | 1.5 (0.7) | 1.3 (0.6) | 1.7 (0.8) | 0.001 | 0.273 |
| LDL-C (mmol/l) | 2.5 (0.8) | 2.6 (0.8) | 2.5 (0.8) | 0.529 | 2.5 (0.8) | 2.4 (0.7) | 2.5 (0.8) | 0.477 | 0.363 |
| UA (umol/l) | 324 (94.9) | 333 (97.3) | 318 (93.0) | 0.129 | 306 (84.7) | 310 (93.3) | 304 (77.6) | 0.644 | 0.028 |
| CEA (ng/ml) | 2.4 (1.5) | 2.2 (1.5) | 2.5 (1.5) | 0.119 | 2.3 (1.6) | 2.18 (1.6) | 2.3 (1.7) | 0.523 | 0.600 |
| CA199 (U/ml) | 12.3 (16.4) | 10.7 (9.2) | 13.5 (20.0) | 0.065 | 11.6 (12.8) | 11.8 (11.8) | 11.4 (13.5) | 0.828 | 0.589 |

Data were expressed as n (%) or mean (standard deviation). NAG, non-atrophic gastritis; GPL, gastric precancerous lesions; p, p-value; BMI, body mass index; H. pylori, helicobacter pylori; CRP, C-reactive protein; TG, triglyceride; LDL-C, low-density lipoprotein-cholesterol; UA, uric acid; CEA, carcinoembryonic antigen; CA199, carbohydrate antigen199.

p# for difference between the NAG and GPL groups in the Derivation and validation cohorts, respectively.

p* for difference between the Derivation cohort and validation cohort.

Table 2 Univariate and multivariate logistic regression analysis of risk factors for GPL based on the development cohort.

| Variables | Univariate analysis | | | | Multivariate analysis | | | |
| --- | --- | --- | --- | --- | --- | --- | --- | --- |
|  | Coef. | OR | 95%CI | p | Coef. | OR | 95%CI | p |
| Female (ref. Male) | -0.156 | 0.856 | 0.568-1.291 | 0.458 |  |  |  |  |
| Age, years | 0.038 | 1.038 | 1.020-1.057 | <0.001 | 0.039 | 1.040 | 1.021-1.060 | <0.001 |
| BMI (kg/m^2^) | 0.141 | 1.152 | 1.080-1.233 | <0.001 | 0.129 | 1.138 | 1.063-1.222 | <0.001 |
| H. pylori (ref. No) | -0.010 | 0.990 | 0.634-1.553 | 0.965 |  |  |  |  |
| Diabetes (ref. No) | 0.421 | 1.523 | 0.817-2.942 | 0.195 |  |  |  |  |
| Hypertension (ref. No) | 0.140 | 1.151 | 0.746-1.785 | 0.529 |  |  |  |  |
| Tobacco use (ref. No) | 0.639 | 1.894 | 1.220-2.976 | 0.005 | 0.447 | 1.563 | 0.978-2.519 | 0.063 |
| Alcohol use (ref. No) | 0.329 | 1.390 | 0.870-2.246 | 0.173 |  |  |  |  |
| CRP (mg/l) | 0.029 | 1.029 | 1.002-1.068 | 0.074 |  |  |  |  |
| TG (mmol/l) | 0.533 | 1.704 | 1.307-2.291 | <0.001 | 0.495 | 1.640 | 1.258-2.199 | <0.001 |
| LDL-C (mmol/l) | -0.082 | 0.922 | 0.715-1.187 | 0.527 |  |  |  |  |
| UA (umol/l) | -0.002 | 0.998 | 0.996-1.000 | 0.127 |  |  |  |  |
| CEA (ng/ml) | 0.107 | 1.113 | 0.975-1.281 | 0.122 |  |  |  |  |
| CA199 (U/ml) | 0.018 | 1.018 | 1.000-1.041 | 0.101 |  |  |  |  |

Coef., coefficient; OR, Odds Ratio; 95%CI, 95% Confidence Interval; ref., reference.


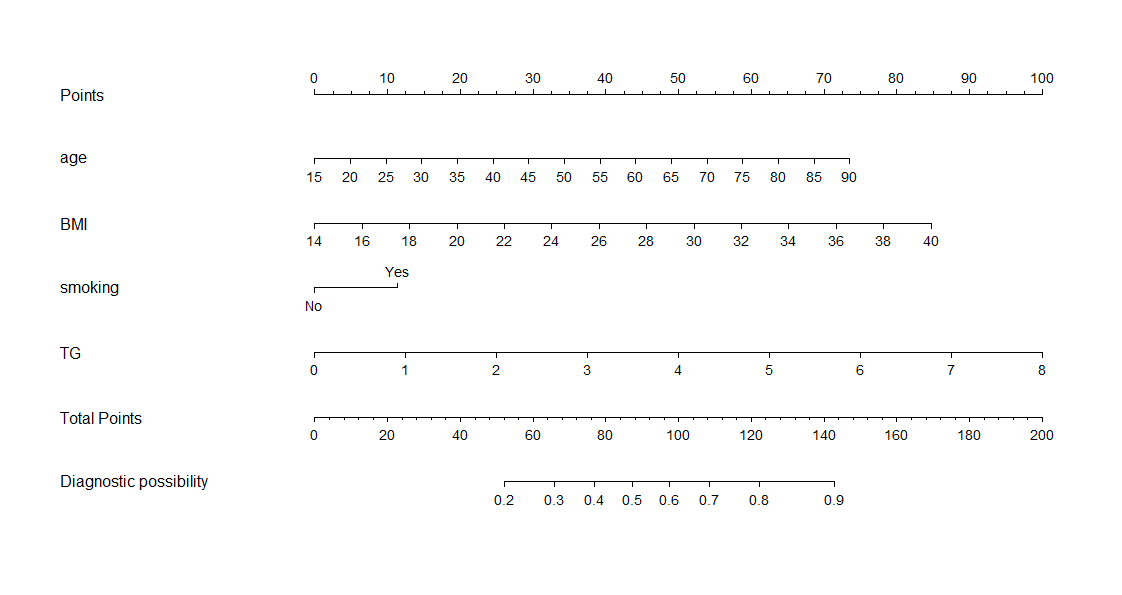


Fig. 1. Nomogram model for Predicting GPL Risk. This nomogram model was designed to predict the risk of GPL based on age, BMI, smoking, and TG. Each categorical variable is assigned a score ranging from 0 to 100. To obtain the total score for predicting GPL, the scores corresponding to each categorical variable are summed up. The total score is then used to determine the probability of categorical outcomes, with different total scores corresponding to different probabilities.

A


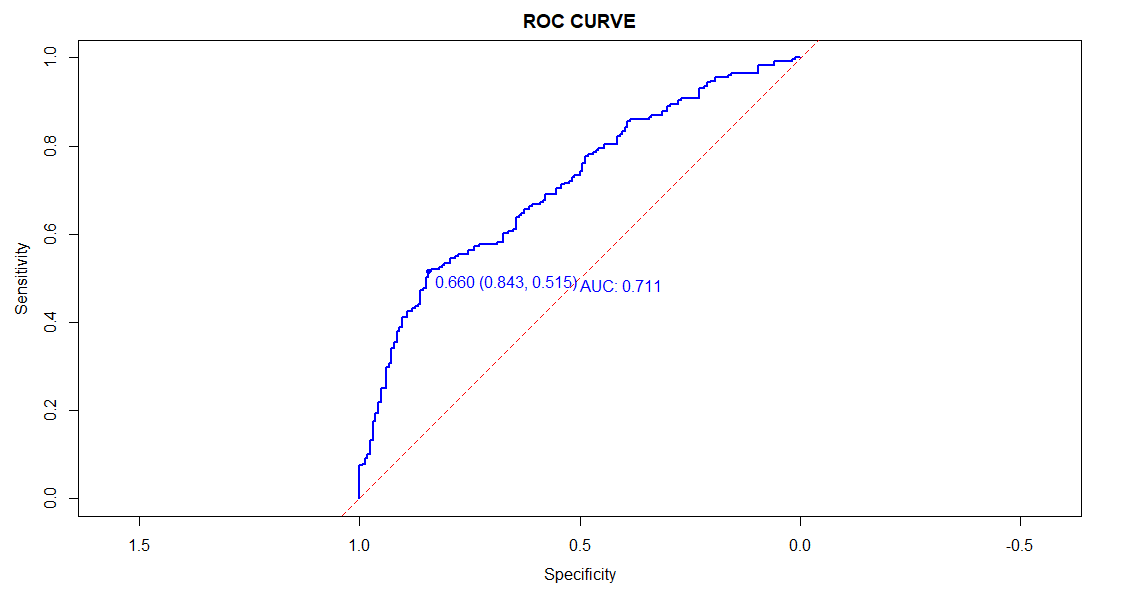


B


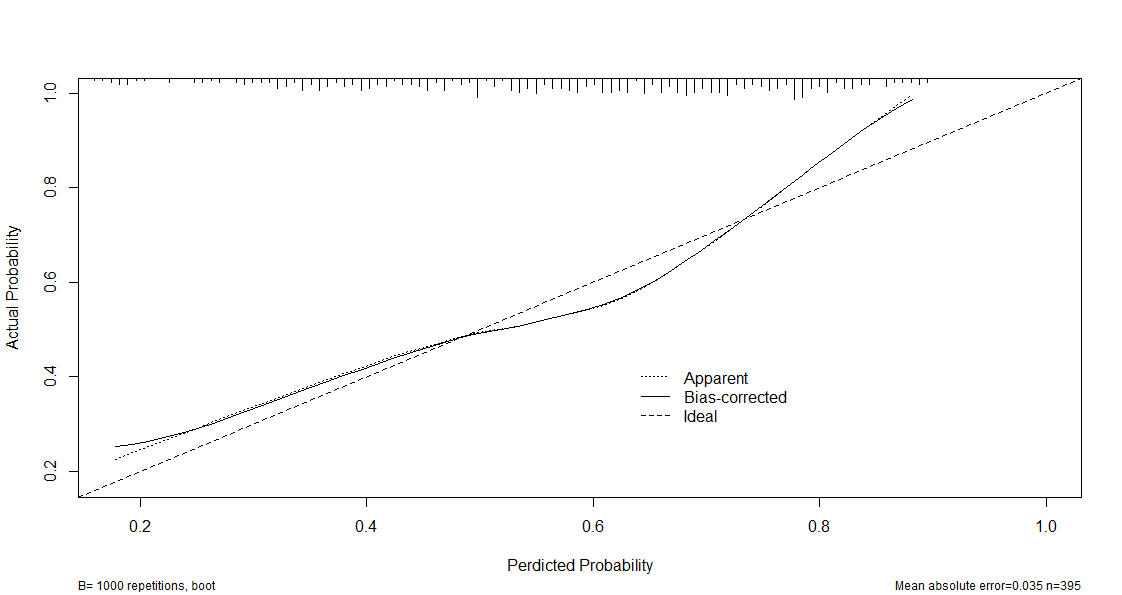


C


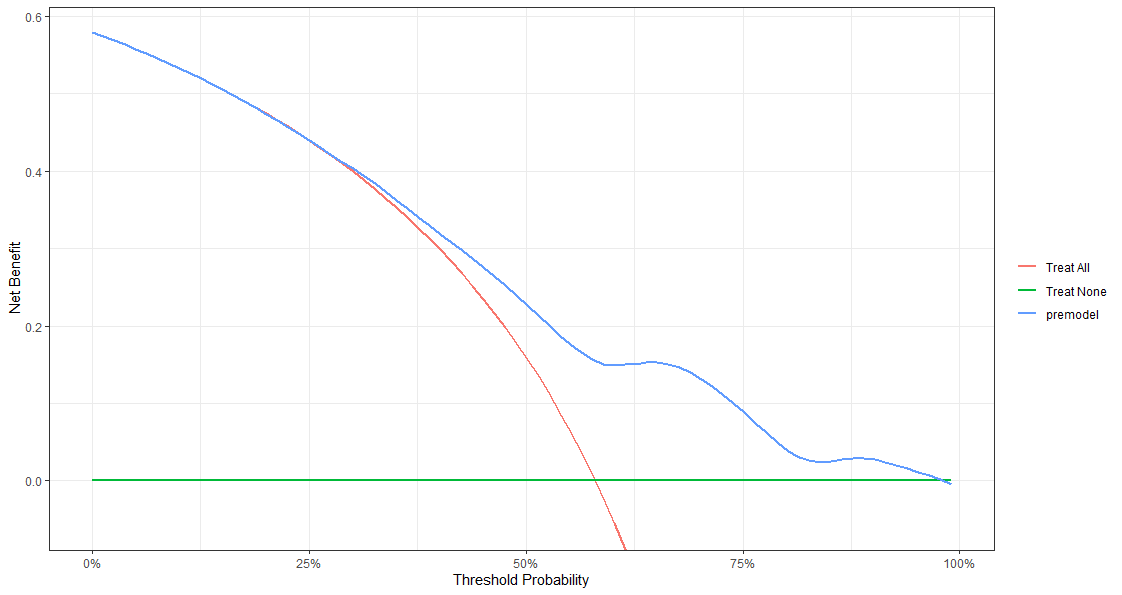


D


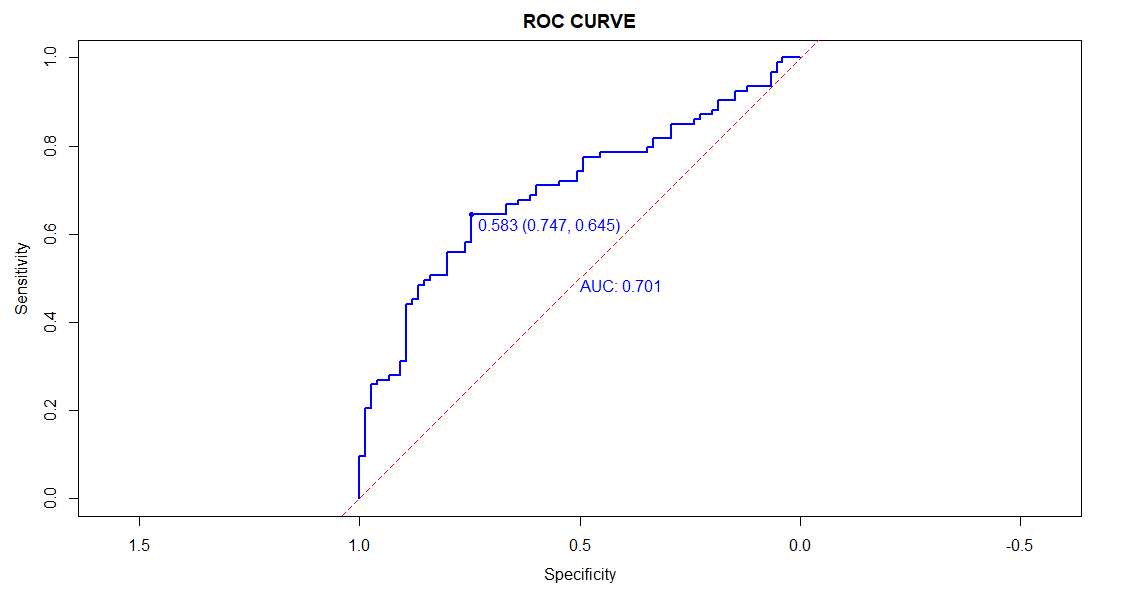


E


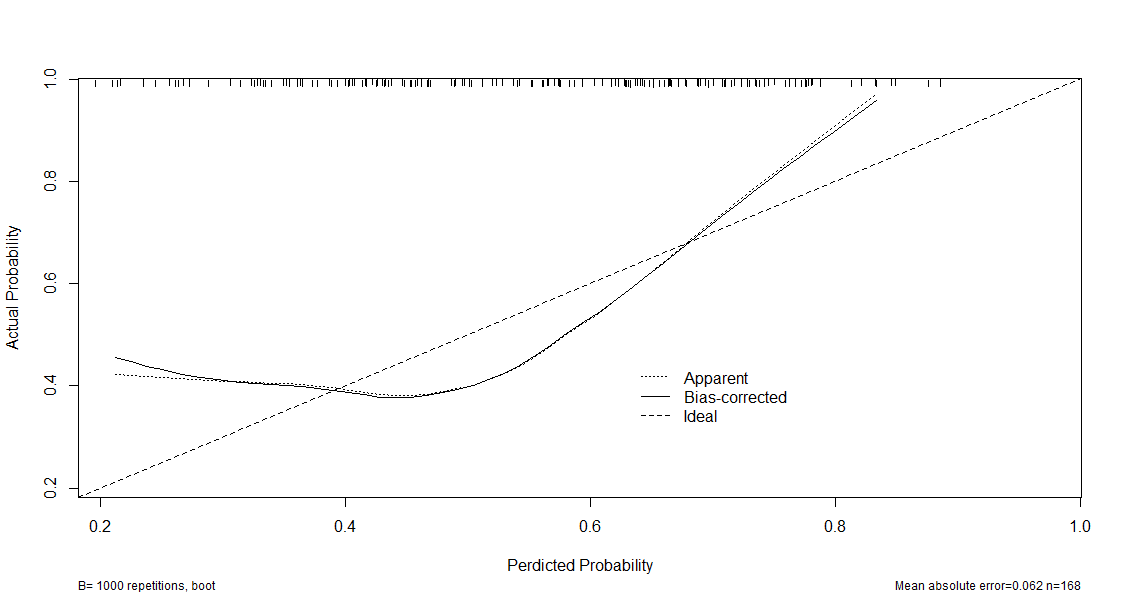


F


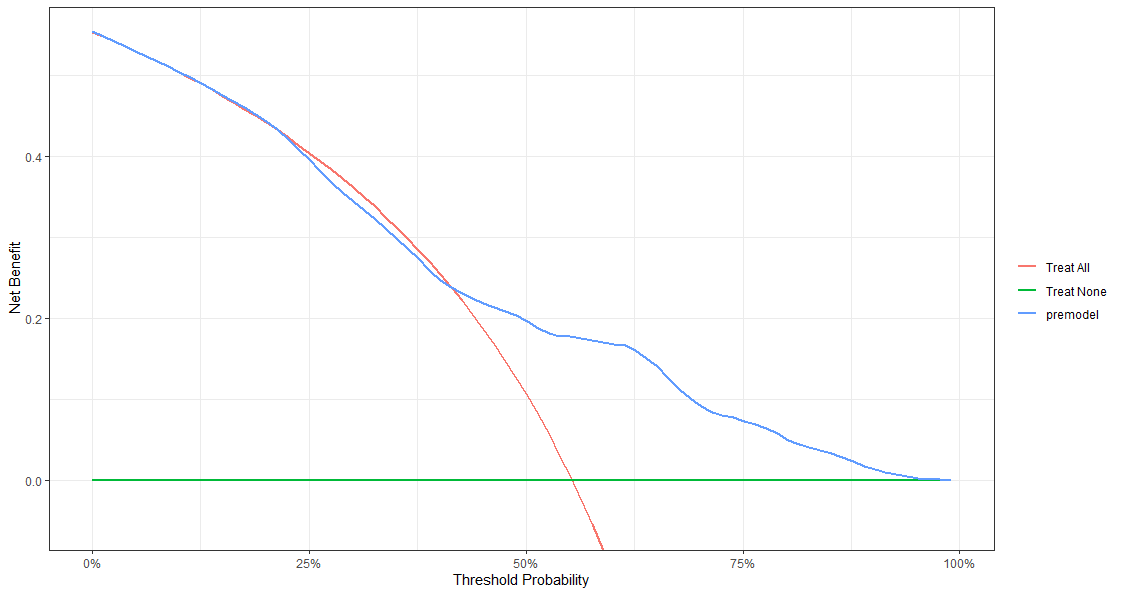


Fig. 2. ROC curves, calibration plots, DCA, and DeLong's test of the prediction model in the derivation cohort and validation cohort. (A) and (D), (B) and (E), (C) and (E) represent the ROC curve, calibration curve, and DCA in the derivation and validation cohort, respectively. Calibration plots showed ideal (100 % agreement) curves, the apparent (actual) and bias-corrected (adjusted) (with 1000 resampling bootstrap). The y-axis represents the actual probability of the GPL model, and the x-axis represents the predicted probability of the GPL model. Clinical decision curve: the y-axis represents the net benefit of GPL, while the x-axis represents threshold probability of GPL. The green line stands for the net benefit for the predict-none-patients as GPL, the red line stands for the net benefit for the predict-all-patients as GPL, and the blue line represents net benefit for the GPL model.
